# Pleural Empyema in Spain (2016–2022): A Nationwide Study on Trends in Hospitalizations, Mortality, and Impact of Comorbidities

**DOI:** 10.3390/jpm15070263

**Published:** 2025-06-20

**Authors:** Begoña Perez-de-Paz, Maria-Jose Fernandez-Cotarelo, Lydia Rodriguez-Romero, Carolina Ribeiro-Neves-Pinto, Natividad Quilez-Ruiz-Rico, Dolores Álvaro-Álvarez, Victor Moreno-Cuerda, Cesar Henriquez-Camacho

**Affiliations:** 1 Department of Pneumology, Hospital Universitario de Mostoles, 28935 Madrid, Spain; begoperezdepaz@gmail.com (B.P.-d.-P.); lrromero@salud.madrid.org (L.R.-R.); natividad.quilez@salud.madrid.org (N.Q.-R.-R.); dolores.alvaro@salud.madrid.org (D.Á.-Á.); 2 Department of Internal Medicine, Hospital Universitario de Mostoles, 28935 Madrid, Spain; mariajose.fernandez.cotarelo@urjc.es (M.-J.F.-C.); vmcuerda@salud.madrid.org (V.M.-C.); 3 Department of Medical Specialties and Public Health, Faculty of Health Sciences, Universidad Rey Juan Carlos, 28922 Madrid, Spain; carolina.nevespinto@gmail.com; 4 Faculty of Medicine, Universidad Francisco de Vitoria, 28223 Madrid, Spain

**Keywords:** pleural empyema, pleural drainage, prognosis, mortality, comorbidity pneumonia, Spain

## Abstract

**Background**: Pleural empyema (PE) is a major cause of morbidity and mortality worldwide. This study aimed to analyze the epidemiological characteristics of patients hospitalized for PE in Spain between 2016 and 2022. **Methods**: This retrospective observational study of PE cases was based on the hospital discharge records from the National Health System between 2016 and 2022. The variables analyzed were sex, age, comorbidities, discharge diagnoses and procedures, overall severity, whether empyema was a primary or secondary diagnosis, admission to the intensive care unit (ICU), length of stay (LOS), in-hospital mortality, and healthcare costs. **Results**: Between 2016 and 2022, 19864 PE cases were diagnosed in Spain, revealing an overall rate of 0.64 per 1000 hospitalizations, with the exception of a slight decline in 2021. The mean age of the patients with PE was 61 years, and 73.85% were men. Most patients had low comorbidities, with a median Charlson comorbidity index (CCI) of 1.7. Most cases (63%) involved secondary diagnoses (pneumonia, pneumococcal pneumonia, sepsis, COVID, or lung cancer). The in-hospital mortality rate was higher in the secondary diagnosis group than in the primary diagnosis group (13.4% vs. 6.2%, respectively, *p* < 0.001). The factors associated with increased mortality included older age (≥66 years), higher CCI scores, ICU admission, and shorter LOS (<10 days). Conversely, pleural drainage and pneumonia as secondary diagnoses were protective factors. **Conclusions**: PE is an increasingly common pathology in clinical practice, especially in older and frail patients. It is associated with high morbidity and mortality, and its prognosis worsens with age and comorbidities. Therefore, early and appropriate diagnosis and standardized management strategies are required to mitigate the mortality and healthcare costs.

## 1. Introduction

Pleural empyema (PE) is defined as pus in the pleural space (described as a thick, yellowish-white, viscous fluid resulting from serum clotting proteins, cellular debris, and fibrin deposition) or pleural fluid with a positive Gram stain or culture [1]. It was described by Egyptian physicians and Hippocrates as an infection of the pleura in 500 BC [2]. Even today, PE remains a relevant pathology, with a high mortality rate of approximately 10–25% [3].

PE is a continuously evolving pleural infection, with high mortality and morbidity [1]. Its incidence is variable and increasing worldwide, with a rate of 5.98/100,000 population in the United States (US) and 7.75/100,000 population in France [4,5,6].

The most common presentation is pneumonia associated with pleural effusion, which becomes infected and evolves into empyema [7,8]. It is usually complicated in 0.32% of patients [3]. The less frequent causes of PE are bronchogenic carcinoma, esophageal rupture, thoracic trauma, infectious mediastinitis with extension to the adjacent pleura, infectious dissemination, and postsurgical infections [1].

PE may develop in cases of aspiration pneumonia (usually in patients with neurological diseases affecting swallowing), ciliary function, gastroesophageal reflux disease, immunosuppression, cancer, alcohol abuse, and poor oral hygiene [2].

*Streptococcus pneumoniae* is the most common cause of PE [9]. *Staphylococcus aureus* and, to a lesser extent, Gram-negative bacteria such as *Klebsiella pneumoniae* are also common in hospitalized patients. Anaerobes such as *Prevotella, Fusobacterium*, and *Peptostreptococcus* are frequently found in patients with aspiration pneumonia [10,11].

Despite the availability of antibiotics and access to vaccines, PE remains a major complication of pneumonia [12]. Approximately one million patients are hospitalized in the US each year, and 5100 are hospitalized annually for PE in France [5]. Of those hospitalized, 20–40% have parapneumonic effusion, 5–10% of these progress to PE, and there is a mortality rate of approximately 16.1% in patients over 65 years of age [13,14].

Current therapies for PE include antibiotics, pleural decortication, and pleural drainage through an endopleural tube with or without the use of thrombolytics [13]. It is important to note that in the surgical management of empyema, pleural decortication is often the only component of a more extensive procedure that also includes thorough drainage and debridement of the pleural cavity. Even with chest tube drainage in surgical settings, treatment fails in up to one-third of the cases [15]. In these situations, surgical treatment with video-assisted thoracoscopy or open drainage of the cavity is required [16].

The economic costs associated with PE are high, reaching 500 million dollars in the US or EUR 21,000 in France per case. This is due to the need for pharmacological and interventional treatments, in addition to hospital stay [14].

The diagnostic and treatment strategies for PE are well illustrated in the medical literature, reflecting their importance. This study aimed to analyze the epidemiology of PE in Spain between 2016 and 2022, as well as patient characteristics, in-hospital mortality, and related factors.

## 2. Materials and Methods

We conducted a retrospective observational study of patients with PE, based on hospital discharge records from the National Health System, between 2016 and 2022. The Ministry of Health provided anonymized data upon request using the minimum basic data set (MBDS). The MBDS includes variables such as sex, date of birth, primary diagnosis, up to 13 secondary diagnoses at discharge, and up to 20 procedures performed during the hospital stay, which were coded according to the International Classification of Diseases, Tenth Revision, Clinical Modification [ICD-10-CM], ICU admission, department responsible for discharge, length of stay (LOS), in-hospital mortality, and costs. Primary diagnosis refers to the condition considered the main cause of hospital admission. Secondary diagnoses were those that coexisted with the main diagnosis either on admission or thereafter during hospital stay. All admissions with a primary or secondary diagnosis of PE (ICD-10-CM code J86.9) were registered by a nurse coder based on the discharge reports. Our search was based on both primary and secondary diagnoses and included most patients diagnosed with PE.

Comorbidity was assessed by calculating the Charlson Comorbidity Index (CCI) using all diagnoses and procedures recorded at discharge. Specific conditions, such as pneumonia, sepsis, pneumococcal infection, lung cancer, and COVID-19 infection, were also recorded. LOS and in-hospital mortality rates were calculated from admission and discharge dates. Severity in each admitted patient was calculated using the All Patient Refined Diagnosis-Related Groups (APR-DRG) Severity of Illness (SOI) level. It was assigned one of four levels of severity: low or minor (1), moderate (2), high or major (3), and extreme (4). The SOI was determined using the 3M grouper software, which uses primary diagnosis, secondary diagnosis (comorbidities and complications), age, procedures performed, and discharge status. Admission costs were calculated using the APR-GRD weights.

### 2.1. Statistical Analysis

Statistical analysis was performed using Stata Statistical Software Release 18. Quantitative variables were expressed as means and standard deviations (SDs), and qualitative variables were expressed as percentages. The distribution of quantitative variables was determined using the one-sample Kolmogorov–Smirnov test. A bivariable analysis according to year was performed using the chi-square test for linear trend (proportions), analysis of variance (means), and Kruskal–Wallis (medians), as appropriate. Admission-based incidence rates were estimated per 1000 admissions, according to data from the Spanish National Institute of Statistics. Multivariate logistic regression analysis was performed for variables that were significant in the univariate analysis and associated with in-hospital mortality. Adjusted odds ratios and 95% confidence intervals were calculated. Estimates are expressed as odds ratios (ORs) and 95% confidence intervals (CIs).

### 2.2. Ethics

The study protocol was approved by the Research Ethics Committee of Universidad Rey Juan Carlos (Ref. CEI: 121220240072025). The investigators received anonymized medical data from the MBDS provided by the Spanish Ministry of Health, and no informed consent was obtained.

## 3. Results

**Figure 1:**
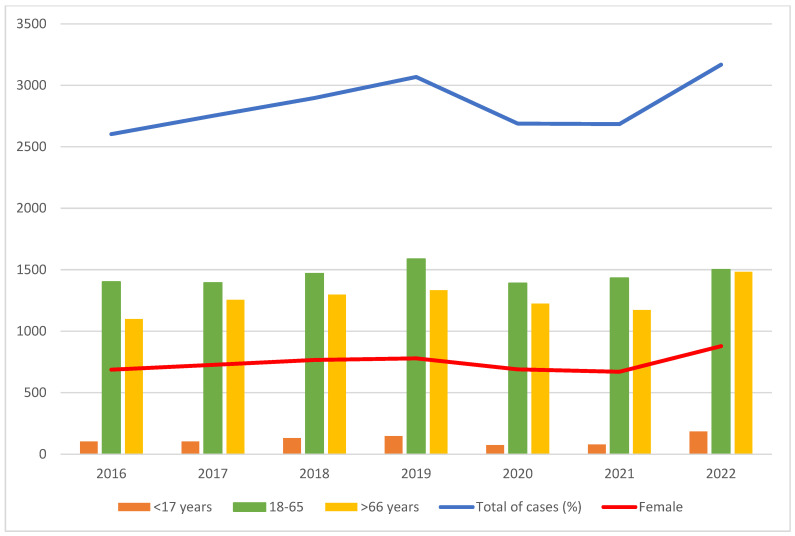
Global rate of empyema cases.

A total of 19,864 patients were diagnosed with PE between 2016 and 2022. The overall rate was 0.64 per 1000 hospitalizations, increasing steadily from 2016 to 2022, except for a slight decrease in 2021 (Table 1). The hospitalization rate for PE has increased by 20.3%, from 0.59 per 1000 admissions in 2016 to 0.71 in 2022. The number of PE cases segregated by sex followed the same pattern (Figure 1). More than half of the PE cases were diagnosed in patients aged 18–65 years (mean age, 61 years), and almost three in four patients were men. In 2022, there was an increase in PE in patients younger than 18 years. Most patients had no or low comorbidity, with a median CCI of 1.7 during the study period, which reached a maximum of 1.8 in 2021.

Most cases (63%) were secondary diagnoses in patients admitted for other reasons. During the study period, 15% of pneumonia, 3.8% of sepsis, and 1% of pneumococcal sepsis cases were reported. The incidence of pneumococcal sepsis, pneumonia, and sepsis peaked in 2018, 2019, and 2022, respectively. A total of 215 COVID-19 cases were collected in 2020, with the highest number of cases occurring in 2021. A total of 214 patients had aspiration, 119 were diagnosed with lung cancer, 54 had tuberculosis, 35 had sepsis due to Group A beta-hemolytic streptococcal infection, and 7 were co-infected with the influenza virus. A total of 9114 patients (91%) required pleural drainage, and its use has increased from 2016 to 2022.

Most patients (74%) were admitted to the medical ward, whereas only 4% required ICU admission. Patients with PE were classified into four grades according to their severity: low (27 cases), moderate (822 cases), high (11,964 cases), and extreme (6993 cases). In all categories, the number of empyema cases increased throughout the study period.

The mean LOS was 20 days, and no significant differences were observed during the study period. The overall in-hospital mortality rate was 11%. The total average cost per hospitalization for PE in Spanish hospitals was 943.44 euros. Costs have increased significantly from 2016 to 2024, peaking in 2020.

### 3.1. Empyema as Primary or Secondary Diagnosis

There were a total of 7360 cases of PE as the primary diagnosis and 12,504 cases as the secondary diagnosis (Table 2). Patients with PE as the primary diagnosis were older (74 vs. 70 years, *p* < 0.001) but had a lower CCI (1.4 vs. 1.8, *p* < 0.001). Significant differences were found between the groups when the data were disaggregated according to CCI: 71% of the patients with primary empyema had a CCI of less than 1, 12% had a CCI of 2, and 17% had a CCI of ≥3. Among the patients with secondary empyema, 63% had a CCI of less than 1, 14% had a CCI of 2, and 23% had a CCI of ≥3.

Patients with PE as the primary diagnosis were more likely to be admitted to the medical ward (81% vs. 64%, *p* < 0.001) and less likely to be admitted to the intensive care unit (ICU) (8% vs. 26%, *p* < 0.001). Differences were also found in mortality between primary and secondary empyema, depending on the service to which the patient was admitted: 369 cases (81%) of primary empyema died in medical services, 46 cases (10%) in surgical services, and 37 cases (8%) in the ICU. Among the deaths due to secondary empyema, 1068 (64%) were managed by medical services, 170 (10%) by surgical services, and 435 (26%) were in the ICU.

Global severity was categorized as high in 66% of patients with PE as the primary diagnosis, whereas most patients with PE as a secondary diagnosis (75%) had extreme severity. In terms of severity of primary cases, 4 patients died with moderate severity empyema, 300 with severe empyema, and 149 with extreme severity empyema. In the secondary cases, there was 1 death among patients with low-severity empyema, 13 among those with moderate-severity empyema, 399 among patients with high-severity empyema, and 1252 among those with extreme-severity empyema.

Pleural drainage was more common in patients with PE as primary diagnosis (86% vs. 79%, *p* = 0.011). The proportion of drainage was higher in the surgical ward (1525 of 1600 patients, 95.3%) than in the medical ward (7412 of 8274 patients, 89.6%). The mean LOS (10 vs. 15 days, *p* < 0.001), in-hospital mortality rate (6.2% vs. 13.4%, *p* < 0.001), and mean cost (912.07 vs. 1030.35 euros, *p* < 0.001) were lower in patients with PE as the primary diagnosis.

The mean age at mortality was 74 years for the primary diagnoses and 70 years for the secondary diagnoses. Patients aged ≥66 years with PE as the primary diagnosis had a higher mortality rate (73%) than those with secondary PE (66%) (*p* = 0.007).

### 3.2. Analysis of In-Hospital Mortality

A low risk of mortality was observed in the age group of 18–65 years when the diagnosis was primary. However, when the diagnosis was secondary or indistinct, this age group was associated with higher mortality (12.30 and 13.45 aOR, respectively). The risk was higher in the age group over 66 years as a secondary diagnosis (aOR, 30.22, CI95% 4.15–219.89, *p* < 0.001). Analyzing the CCI, a higher risk of mortality was observed when the index was more than 3 in the secondary diagnosis (aOR: 4.39, CI95% 3.51–5.49, *p* < 0.001) and in the primary diagnosis (aOR: 3.64, CI95% 2.72–4.87, *p* < 0.001).

In patients with a primary diagnosis of PE, being in charge of surgical services acted as a factor related to a lower risk of mortality (aOR 0.31, CI95% 0.18–0.50, *p* < 0.001), while being in the ICU was associated with a higher mortality (aOR: 6.93, CI95% 2.86–16.75, *p* < 0.001).

No influence was observed on mortality due to COVID-19 as the origin of secondary empyema or any other type of empyema. In patients with pneumonia, decreased mortality was noted when secondary empyema or any other type of empyema was present (Table 3). Lung cancer increased mortality in patients with secondary (aOR, 2.46, CI95% 1.32–4.58, *p* = 0.005) or any type of diagnosis (aOR, 2.93, CI95% 1.58–5.44, *p* = 0.001). Sepsis increased mortality in patients with secondary diagnosis or any type of diagnosis (aOR, 1.74, CI95% 1.27–2.38, *p* < 0.001). Pleural drainage decreased mortality in all of the groups studied.

When assessing severity, the only value that has been observed to significantly decrease mortality is when severity is classified as high in patients diagnosed with primary empyema. When assessing the LOS of the patients, an increase in mortality was observed in all groups studied when it was less than 10 days.

## 4. Discussion

This study analyzed the characteristics of PE in hospitals in Spain and the trend in recent years, showing a progressive increase. This nationwide study provides critical insights into the epidemiology, clinical outcomes, and economic burden of this disease. To the best of our knowledge, there is no published big data on PE cases in Spain. Our findings demonstrate a steady rise in the incidence of PE, with a 20.3% increase in the hospitalization rate. This pattern coincides with that of other studies, reflecting a slight increase over time [3,11,12,13,14,17]. Despite medical advancements, PE continues to have a high in-hospital mortality rate (11%). Importantly, patients with PE as a secondary diagnosis had notably worse outcomes than those with PE as a primary diagnosis, including higher comorbidity burden, longer hospital stay, increased healthcare costs, and more than double the mortality rate (13.4% vs. 6.2%).

The increasing trend in PE incidence aligns with global patterns and may be attributed to improved diagnostic capabilities, particularly with the wider use of thoracic ultrasound [13,18], rising antimicrobial resistance, and an aging population. The temporary decline in 2021 appears to be linked to disruptions caused by the COVID-19 pandemic, followed by a sharp rebound the following year. A significant increase in mortality among the extremes of age (<17 years and >66 years) during this period has also been demonstrated, with mortality values consistent with those of previous studies [14,19].

This also supports the hypothesis that there is an increase in the elderly population, which is more susceptible to complications from respiratory infections and comorbidities.

Finally, this increase may be related to increased diagnoses because more lung ultrasounds are currently performed in medical services, resulting in more pleural effusions being detected and diagnosed as empyema, which could otherwise be treated as pneumonia.

Comorbidity burden, measured by the Charlson Comorbidity Index (CCI), was a key predictor of mortality across diagnostic categories. Patients with higher CCI scores (≥3) were significantly more likely to die during hospitalization, regardless of whether PE was a primary or secondary diagnosis. This finding reinforces the value of comorbidity scoring in risk stratification. Other studies, such as that of Ho et al., which used CCI to relate it to COPD, pneumonia, or empyema, used a different categorization of the index based on the age-adjusted CCI [20]. Although they proposed a different categorization of indices, we used the simplest one. Nevertheless, we can still conclude, as do Asai et al. and Ho et al., that the higher the CCI, the higher is the risk of mortality due to PE [3,20]. A more standardized categorization of this index should be used to make better comparisons and obtain more reliable conclusions.

Sepsis and lung cancer were independently associated with increased mortality in patients with secondary PE. It occurs as a complication secondary to lung cancer in 0.1–7.9% of cases. It can be caused by iatrogenesis by inducing infection in the pleural space after repeated thoracentesis or immunosuppression. It can also appear secondary to bronchial obstruction by a mass, causing pneumonia and its subsequent complication of PE, by rupture of a cavitary carcinoma as the disease progresses, or by tumor necrosis after chemotherapy. PE can also appear after transbronchial biopsy in patients with suspected lung cancer [21]. All of these comorbidities confer greater fragility to the patient and align with the results of our study, which reflect higher mortality in cases presenting with PE as a secondary diagnosis after lung cancer. These data are consistent with those of other studies, indicating that PE tends to be present in fragile patients [5].

Conversely, pneumonia-related PE is associated with reduced mortality, likely because of earlier clinical recognition and management. These patterns suggest that early diagnosis and intervention are critical for improving outcomes, especially in patients with complex underlying conditions [4,22]. These patients were not as frail as those with PE secondary to sepsis, who had a significantly higher mortality rate in this study.

COVID-19 has been a cause of PE since its appearance in 2020, with an annual trend toward increasing cases of PE secondary to COVID-19. We did not obtain significant results regarding the relationship between COVID-19 and empyema mortality, likely because of the small number of cases. Chan et al. reported non-significant results for mortality before and after COVID-19 [17].

Since 2016, we have observed that patients with PE classified as mild, low, or medium severity have increased in line with the rise in overall cases but decreased in percentage terms. The mean LOS for patients diagnosed with PE has not increased since 2016 but is higher than that of other populations in previous reports [23]. Additionally, we noted that in-hospital mortality and the mean cost of care for these patients increased and that these data are in accordance with the literature. In terms of cost, this may be influenced by the greater availability of diagnostic mechanisms, techniques, and treatments over the years [14,23]. Some studies, such as Bobbio et al., reported a mean LOS of 21 days for patients with PE, whereas in our study, it was 10 days for the primary diagnosis and 15 days for the secondary diagnosis, which may reflect an improvement in patient management [5]. Furthermore, our analysis showed that patients with a LOS shorter than 10 days had a higher risk of mortality than those admitted for more than 10 days, regardless of the type of diagnosis. This could be because several patients died prematurely due to sepsis, either because of PE or their comorbidities. Further analysis would help to clarify this finding.

The need for pleural drainage increased throughout the study period and was associated with lower mortality in all diagnostic categories, supporting its continued use as a first-line therapy. This aligns with existing evidence that timely pleural drainage reduces complications and improves prognosis. According to the American Association for Thoracic Surgery consensus guidelines for the management of PE, 30% of the cases require drainage [13]. In this study, we have been able to verify how the use of pleural drainage has increased over the years in accordance with the literature published in 2017 by Redden et al. [22]. The authors suggested the use of pleural drainage (and even better, drainage by video-assisted thoracoscopic surgery or VATS, as it decreases LOS) to reduce mortality in patients with PE. Our study showed that drainage is a protective factor against mortality, regardless of whether PE is primary or secondary. Furthermore, some authors have proposed that the earlier the drainage is placed (before the first four weeks from the onset of symptoms), the better the patient’s prognosis [24].

When performing multivariate analysis based on whether the diagnosis was primary or secondary, several relevant results were obtained. Our study showed that patients with primary PE admitted to surgical services had a lower risk of mortality, which supports the key finding that early surgical treatment helps minimize complications, as reported in other studies [16,22]. However, Semmelmann et al. indicated that these surgeries should be performed with caution and only in selected patients, owing to the associated risks [25]. Other studies suggest that using fibrinolytics is preferable and that simple thoracentesis yields better results in terms of average LOS than drainage tubes [26,27]. The European Respiratory Society recommends that further studies be conducted to standardize the management of PE and to determine why this lower mortality risk has not been significantly observed when the diagnosis is secondary or when we have not made a distinction [28].

As previously mentioned, patients admitted to the ICU are at much higher risk of in-hospital mortality. We believe that this difference is due to the fact that patients who are candidates for these units already have a very poor baseline condition or a series of comorbidities that make the prognosis unfavorable, regardless of their management.

Finally, the cost associated with PE has also increased, rising from 912.07 euros per hospitalization in 2016 to 1030.35 euros in 2022 (13% cost increase). This parallels the increase in LOS and severity. This reflects both the growing clinical complexity of PE cases and the resource intensity of their management. An increase in the number of extremely severe cases and an increase in LOS were likely contributors. Cost-effectiveness analyses of preventive strategies, such as pneumococcal vaccination in high-risk adults, are needed.

Limitations include the retrospective design using administrative hospital discharge records (potential coding errors by non-physician professionals) and lack of treatment-detailed data (diagnostic methods, antibiotic regimens, and surgical procedures). Therefore, misclassification or selection bias is possible. Another limitation is that CCI categorization may oversimplify comorbidity burdens compared with studies that use age-weighted scores. Prospective studies should assess whether early surgical referral or adjunctive therapies (e.g., fibrinolytics) improve outcomes in patients with secondary PE. Comparative cost analyses of drainage modalities (e.g., VATS vs. tube thoracostomy) are also needed.

## 5. Conclusions

Pleural empyema remains a significant clinical and economic challenge in Spain, with mortality heavily influenced by comorbidities and diagnostic context. Its prevalence has increased in recent years and primarily affects older and frail patients with comorbidities. Early diagnosis and appropriate treatment with pleural drainage reduce patient mortality, supporting the current guidelines advocating early intervention. Patients diagnosed with PE secondary to other pathologies had more comorbidities, longer LOS, and higher associated health care costs. Further studies on PE should be conducted to analyze other protective factors. 

## Figures and Tables

**Table 1:** Characteristics of empyema cases in patients hospitalized in the National Health System, 2016–2022.

		2016	2017	2018	2019	2020	2021	2022	Total	*p*-Value
Total PE cases		2604	2752	2897	3068	2689	2685	3169	19,864	
Total admission		4,394,207	4,562,182	4,529,107	4,560,089	4,032,912	4,316,158	4,485,352	30,880,007	
PE per 1000 admissions		0.59	0.60	0.64	0.67	0.67	0.62	0.71	0.64	<0.001
Female sex (%)		687 (26.4)	726 (26.4)	766 (26.4)	779 (25.4)	689 (25.6)	670 (25)	878 (27.7)	5195 (26.15)	0.274
Mean age (SD)		60 (19)	61 (19)	60 (20)	60 (20)	61 (18)	61 (18)	61 (21)	61 (19)	0.033
Age group (%)	≤17	104 (4)	104 (3.8)	131 (4.5)	148 (4.8)	75 (2.8)	80 (3)	185 (6)	827 (4.2)	<0.001
	18–65	1401 (53.8)	1393 (50.6)	1468 (50.7)	1586 (51.7)	1389 (51.7)	1432 (53.3)	1501 (47.4)	10170 (51.2)	
	≥66	1099 (42.2)	1255 (45.6)	1298 (44.8)	1334 (43.5)	1225 (45.6)	1173 (43.7)	1483 (46.8)	8867 (44.6)	
PE as primary diagnosis (%)		1039 (40)	1056 (38)	1064 (37)	1106 (36)	986 (37)	969 (36)	1140 (36)	7360 (37)	0.017
CCI categories (%)	≤1	1754 (67)	1824 (66)	1972 (68)	2010 (66)	1761 (66)	1688 (63)	2059 (65)	13068 (66)	0.006
	2	362 (14)	371 (14)	360 (12)	399 (13)	353 (13)	388 (14)	417 (13)	2650 (13)	
	≥3	488 (19)	557 (20)	565 (20)	659 (21)	575 (21)	609 (23)	693 (22)	4146 (21)	
Mean CCI (SD)		1.6 (2.5)	1.6 (2.5)	1.6 (2.4)	1.7 (2.6)	1.7 (2.5)	1.8 (2.6)	1.7 (2.5)	1.7 (2.5)	<0.001
Pneumonia		412 (15.8)	445 (16.2)	479 (16.5)	471 (15.4)	369 (13.7)	337 (12.6)	496 (15.7)	3009 (15.2)	<0.001
Sepsis		76 (3)	113 (4)	105 (3.6)	135 (4.4)	97 (3.6)	100 (3.7)	123 (4)	749 (3.8)	0.129
Pneumococcal sepsis		25 (1)	19 (0.7)	42 (1.5)	50 (1.6)	22 (0.8)	16 (0.6)	35 (1.1)	209 (1)	<0.001
COVID-19		-	-	-	-	40 (1.5)	88 (3.2)	87 (2.7)	215 (1)	<0.001
Pleural drainage (%)		1262 (88)	1315 (88)	1374 (91)	1427 (91)	1236 (92)	1167 (93)	1333 (93)	9114 (91)	<0.001
Lung cancer		21 (0.8)	24 (0.9)	12 (0.4)	19 (0.6)	10 (0.4)	15 (0.6)	18 (0.6)	119 (0.6)	0.149
Hospital ward (%)	Medical	1869 (72)	2026 (74)	2190 (76)	2296 (75)	2021 (75)	1991 (74)	2366 (75)	14759 (74)	0.009
	Surgical	637 (24)	618 (22)	592 (20)	677 (22)	573 (21)	570 (21)	690 (22)	4357 (22)	
	ICU	96 (4)	100 (4)	112 (4)	90 (3)	90 (3)	112 (4)	103 (3)	703 (4)	
	Others	2 (0.1)	8 (0.3)	3 (0.1)	5 (0.2)	11 (0.4)	11 (0.4)	7 (0.2)	40 (0.2)	
Severity	Low	3 (0.1)	4 (0.2)	3 (0.1)	6 (0.2)	2 (0.1)	4 (0.1)	5 (0.2)	27 (0.1)	<0.001
	Moderate	130 (5)	127 (4.6)	100 (3.5)	120 (3.9)	108 (4)	95 (3.5)	142 (4.5)	822 (4)	
	High	1673 (64)	1655 (60)	1797 (62)	1893 (62)	1569 (58)	1470 (55)	1907 (60)	11964 (60)	
	Extreme	778 (30)	954 (34.7)	989 (34)	1040 (34)	1007 (37)	1112 (41)	1113 (35)	6993 (35)	
Mean LOS (SD) days		19.7 (20)	20.2 (23)	19.6 (20)	20.2 (24)	20.6 (21)	20.8 (25)	20 (23)	20 (22)	0.074
In-hospital mortality (%)		287 (11)	289 (10.5)	283 (9.8)	304 (9.9)	286 (10.6)	333 (12.4)	350 (11)	2132 (11)	0.035
Mean cost (EUR)		886.53	876.92	920.78	909.94	1083.88	995.10	938.18	943.44	<0.001

Abbreviations: PE, pleural empyema; CCI, Charlson comorbidity index; ICU, intensive care unit; LOS, length of stay; SD, standard deviation; COVID-19: Coronavirus disease.

**Table 2:** Comparison of patients with PE as primary or secondary diagnosis.

		Primary Diagnosis N (%)	Secondary Diagnosis N (%)	*p*-Value
Total		7360	12504	
Year	2016	68 (15)	219 (13)	0.809
	2017	60 (13)	229 (14)	
	2018	61 (13)	222 (13)	
	2019	62 (14)	242 (14)	
	2020	53 (12)	233 (14)	
	2021	76 (17)	257 (15)	
	2022	73 (16)	277 (17)	
Female sex		116 (26)	454 (27)	0.541
Mean age (SD)		74 (13)	70 (14)	<0.001
Age group (%)	≤17	-	9 (0.5)	0.007
	18–65	123 (27)	567 (34)	
	≥66	330 (73)	1103 (66)	
Mean CCI (SD)		1.4 (2.3)	1.8 (2.6)	<0.001
Charlson commorbidity index (%)	≤1	5204 (71)	7864 (63)	<0.001
	2	908 (12)	1742 (14)	
	≥3	1248 (17)	2898 (23)	
Hospital ward (%)	Medical	369 (81)	1068 (64)	<0.001
	Surgical	46 (10)	170 (10)	
	ICU	37 (8)	435 (26)	
	Others	1 (0.2)	6 (0.4)	
Pneumonia (%)		-	3369 (27)	
Sepis (%)		-	749 (6)	
Lung abscess (%)		-	253 (2)	
Lung Cancer (%)		-	220 (2)	
COVID-19 (%)		-	215 (2)	
Pneumococcal bacteremia (%)		-	209 (1.6)	
COPD (%)		-	161 (1)	
Aspiration pneumonia (%)		-	143 (1)	
Pleural drainage (%)		242 (86)	487 (79)	0.011
Severity (%)	Low	**-**	1(0.1)	<0.001
	Moderate	4 (1)	13(1)	
	High	300 (66)	399 (24)	
	Extreme	149 (33)	1252 (75)	
Mean LOS (SD)		10 (4–19)	15 (6–30)	<0.001
Mortality		453 (6.2)	1679 (13.4)	<0.001
Mean cost (EUR)		912.07 (363.7)	1030.35 (436.7)	<0.001

Abbreviations: CCI, Charlson comorbidity index; ICU, intensive care unit; LOS, length of stay; COVID-19, Coronavirus disease.

**Table 3:** Multivariate analysis of factors associated with in-hospital mortality in patients with PE.

		In-Hospital Mortality		
		Primary Diagnosis	Secondary Diagnosis	Any Position
		aOR (95%CI) *p* Value	aOR (95%CI) *p* Value	aOR (95%CI)
Year		1.01 (0.94–1.07) 0.751	0.98 (0.93–1.03) 0.592	0.99 (0.95–1.03) 0.831
Female sex		1.26 (0.94–1.69) 0.111	1.18 (0.94–1.47) 0.137	1.20 (1.01–1.44) 0.034
Age	≤17 (reference)	1	1	1
	18–65	0.34 (0.25–0.46) <0.001	12.30 (1.68–89.56) 0.013	13.45 (1.85–97.29) 0.010
	≥66	-	30.22 (4.15–219.89) 0.001	34.74(4.80–251.01) <0.001
CCI	≤1 (reference)	1	1	1
	2	2.13 (1.49–3.05) <0.001	2.52 (1.91–3.32) <0.001	2.40 (1.93–2.98) <0.001
	≥3	3.64 (2.72–4.87) <0.001	4.39 (3.51–5.49) <0.001	4.22 (3.54–5.03) <0.001
Hospital ward	Medical	-	0.71 (0.13–3.74) 0.688	0.77 (0.15–3.89) 0.752
	Surgical	0.30 (0.18–0.50) <0.001	0.32 (0.05–1.77) 0.194	0.29 (0.05–1.52) 0.145
	ICU	6.93 (2.86–16.75) <0.001	9.38 (1.70–51.68) 0.010	9.29 (1.77–48.82) 0.008
COVID-19		-	1.57 (0.67–3.69) 0.296	1.61 (0.68–3.77) 0.270
Pneumonia		-	0.63 (0.47–0.84) 0.002	0.67 (0.51–0.89) 0.006
Lung cancer		-	2.46 (1.32–4.58) 0.005	2.93 (1.58–5.44) 0.001
Sepsis		-	1.68 (1.22–2.31) 0.001	1.74 (1.27–2.38) <0.001
Pleural drainage		0.52 (0.35–0.77) 0.001	0.46 (0.35–0.60) <0.001	0.45 (0.37–0.56) <0.001
Severity	Low (reference)	1	1	1
	Moderate	-	0.82 (0.17–3.84) 0.807	0.49 (0.11–2.25) 0.366
	High	0.24 (0.17–0.33) <0.001	0.55 (0.15–2.01) 0.347	0.43 (0.12–1.56) 0.204
	Extreme	-	1.45 (0.40–5.22) 0.568	1.40 (0.39–5.05) 0.602
LOS	<10 days	2.67 (2.03–3.49) <0.001	2.34 (1.90–2.89) <0.001	2.40 (2.04–2.83) <0.001
	≥10 days (reference)	1	1	1

Abbreviations: ORa, adjusted odds ratio; CCI, Charlson comorbidity index; ICU, intensive care unit; COVID-19, Coronavirus disease; IBD, inflammatory bowel disease; LOS, length of stay.

## Data Availability

The dataset used and analyzed in the current study is available from the corresponding author upon reasonable request.
